# Systemic corazonin signalling modulates stress responses and metabolism in *Drosophila*

**DOI:** 10.1098/rsob.160152

**Published:** 2016-11-03

**Authors:** Olga I. Kubrak, Oleh V. Lushchak, Meet Zandawala, Dick R. Nässel

**Affiliations:** Department of Zoology, Stockholm University, 10691 Stockholm, Sweden

**Keywords:** corazonin receptor, neuropeptide, fat body, stress signalling, insulin-like peptides

## Abstract

Stress triggers cellular and systemic reactions in organisms to restore homeostasis. For instance, metabolic stress, experienced during starvation, elicits a hormonal response that reallocates resources to enable food search and readjustment of physiology. Mammalian gonadotropin-releasing hormone (GnRH) and its insect orthologue, adipokinetic hormone (AKH), are known for their roles in modulating stress-related behaviour. Here we show that corazonin (Crz), a peptide homologous to AKH/GnRH, also alters stress physiology in *Drosophila*. The Crz receptor (CrzR) is expressed in salivary glands and adipocytes of the liver-like fat body, and CrzR knockdown targeted simultaneously to both these tissues increases the fly's resistance to starvation, desiccation and oxidative stress, reduces feeding, alters expression of transcripts of *Drosophila* insulin-like peptides (DILPs), and affects gene expression in the fat body. Furthermore, in starved flies, CrzR-knockdown increases circulating and stored carbohydrates. Thus, our findings indicate that elevated systemic Crz signalling during stress coordinates increased food intake and diminished energy stores to regain metabolic homeostasis. Our study suggests that an ancient stress-peptide in Urbilateria evolved to give rise to present-day GnRH, AKH and Crz signalling systems.

## 1. Introduction

Stress can be evoked by a multitude of different environmental factors and animals have evolved an arsenal of mechanisms to respond to such aversive stimuli, both systemically and at the cellular level [1–3]. At the systems level, hormonal and neuronal pathways are involved both in mediating stress signals and in resetting the homeostasis. Thus, in mammals, corticosteroids as well as several neuropeptides and peptide hormones have been identified in stress response pathways [4–11].

The vinegar fly *Drosophila* has emerged as a versatile genetic model for analysis of stress responses, both at the cellular and organismal levels [2,12–17]. At the organismal level, *Drosophila* insulin-like peptides (DILPs) and adipokinetic hormone (AKH), an insect orthologue of mammalian gonadotropin-releasing hormone (GnRH), play important roles in various stress responses and affect longevity [2,18–21]. Corazonin (Crz) is another *Drosophila* peptide ancestrally related to AKH/GnRH, which has been proposed as a stress-induced hormone based on various actions revealed in several insect species [22–27], but mechanisms of Crz function in stress are not known. The Crz receptor (CrzR; CG10698) is evolutionarily related to that of mammalian gonadotropin-releasing hormone (GnRH), but also those of arthropod AKH and AKH-corazonin-like peptide (ACP) [28,29]. GnRH is known to mediate metabolic and stress-related effects on reproduction [10,11,30,31], and thus it may be that an ancestral role of Crz, AKH and GnRH in metabolism and stress has been conserved over evolution in parallel with a diversification of other functional roles [24,25].

We showed earlier that knockdown of Crz in the Crz-producing dorsolateral peptidergic neurons (DLPs) in the *Drosophila* brain affects metabolism and resistance to starvation stress [32]. As the DLPs are neurosecretory cells with axon terminations in the corpora cardiaca, anterior aorta and intestine [32–34], it is likely that Crz primarily functions as circulating hormone acting on peripheral tissues. This is supported by detecting expression of the CrzR in the fat body, salivary glands and heart of adult *Drosophila* [35,36] (see also FlyAtlas, http://flyatlas.org). Thus, we set out to investigate the role of systemic Crz signalling under normal conditions and during stress in flies. We used different fat body Gal4 drivers to knockdown the CrzR and tested the flies in a set of assays for effects on metabolism, stress tolerance, gene expression and neuropeptide levels. We found that these drivers also target expression to the salivary glands, a tissue known to express the *CrzR* [35,36]. Our findings suggest that systemic Crz signalling predominantly to the fat body and salivary glands regulates starvation, desiccation and oxidative stress resistance, as well as food ingestion. Furthermore, in starving flies CrzR-knockdown leads to increased circulating and stored carbohydrates, and altered expression of several genes in the fat body. We also find indications of feedback from the fat body to endocrine cells of the brain and corpora cardiaca as targeted CrzR-knockdown alters *dilp3*, *dilp5* and *Crz* transcript levels and Crz and AKH peptide levels. As a comparison to knockdown of the *CrzR* with the fat body GAL4 drivers, we also targeted *CrzR*-RNAi more broadly with a *CrzR*-Gal4 driver, and furthermore analysed the effects of Crz peptide knockdown (*Crz*-Gal4>UAS-*Crz*-RNAi). These experiments produced similar phenotypes in the assays performed, suggesting that a major role of Crz signalling in stress and food intake is via peripheral targets. Thus, systemic Crz signalling, including the fat body as a target, regulates food intake, carbohydrate metabolism and storage, and affects the expression of *Upd2* in the fat body, which is a feedback signal from the fat body to the brain [37]. Crz may thus operate in stress responses in association with insulin-like peptides and AKH.

## 2. Results

### 2.1. The CrzR is expressed by the adult fat body cells

As there is a sex-dimorphic distribution of Crz-producing cells [38,39], and male stress responses were more strongly affected by genetic manipulations of these cells [24], we performed all our experiments on male flies.

**Figure 1.**
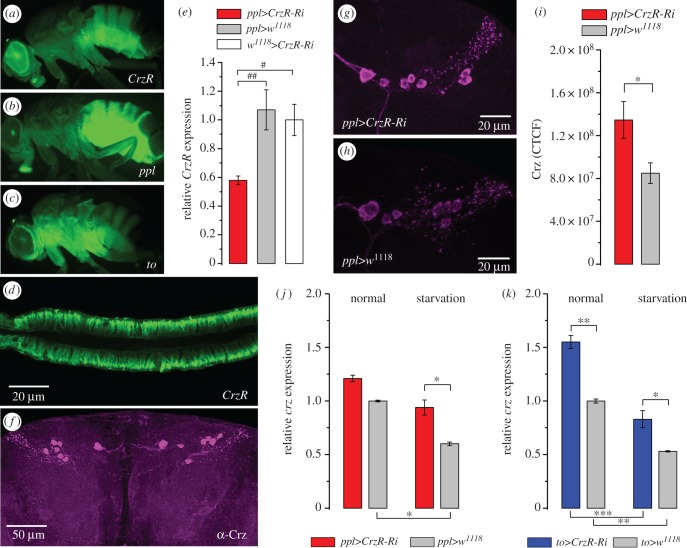
The corazonin receptor (CrzR) is expressed in adipocytes of *D. melanogaster* fat body and its knockdown leads to a compensatory increase of corazonin peptide (Crz) and transcript (*crz*) expression. (*a*) The CrzR is expressed in the adult fly fat body (*CrzR*>GFP), mainly in the abdomen. (*b*,*c*) Two different Gal4 drivers (*ppl*-Gal4 and *to*-Gal4) display GPF expression in adult flies. These were used for targeting UAS-*CrzR*-RNAi in subsequent experiments. In electronic supplementary material, figure S1*a–d*, we show details of GFP expression, as well as GFP expression in other tissues. (*d*) Expression of CrzR in salivary gland revealed by CrzR-Gal4>UAS-GFP). (*e*) *CrzR*-RNAi efficiency was tested by qPCR in *ppl*>*CrzR*-RNAi flies (*ppl>CrzR-Ri*). The efficiency for the CrzR-RNAi driven by an *actin*-Gal4 is shown in electronic supplementary material, figure S1*e*. (*f*) Corazonin-immunolabelling is found in a set of seven dorsolateral peptidergic neurons (DLPs) in each brain hemisphere (shown in *w^1118^* fly). (*g–i*) Knockdown of CrzR in fat body/salivary glands by *ppl*-Gal4 induces an increase of CRZ expression in DLPs. (*j*,*k*) Using *ppl*-Gal4 and *to*-Gal4 to drive *CrzR*-RNAi results in increased *crz* transcript levels, especially after 36 h starvation. Data in graphs are presented as means ± s.e.m., *n* = 3–4 independent replicates with 8–12 flies in each replicate. (*e*) Kruskal–Wallis's test followed by pairwise comparisons using Wilcoxon's rank sum test with ^#^*p* < 0.05, ^##^*p* < 0.01, (*i*) Student's *t*-test with *p < 0.05, **p < 0.01 and (*j*,*k*) ANOVA followed with Tukey's test *p* < 0.05, **p < 0.01, ***p < 0.001.

FlyAtlas transcript expression data suggest that the CrzR (CG10698) is highly expressed in the adult fat body, salivary glands and dorsal vessel [36], and a previous study substantiated the adult-specific expression in the fat body by using a *CrzR*-Gal4 [35]. We confirmed the expression of *CrzR*-Gal4-driven GFP in the fat body in adult flies (figure 1*a*). The receptor distribution coincides with pumpless (*ppl*) [40] and takeout (*to*) [41] GFP expression (figure 1*b*,*c*; electronic supplementary material, figure S1*a*,*b*). It should be noted that the *CrzR*-, *ppl-* and *to*-Gal4 lines also drive GFP expression in the salivary glands (figure 1*d*; electronic supplementary material, figure S1*c*,*d*), and *to-*Gal4 additionally in the proventriculus (electronic supplementary material, figure S1*d*), but neither line displays GFP in the dorsal vessel (heart). After targeting *CrzR*-RNAi to the fat body with the *ppl*-Gal4, we observed a 40% decrease in *CrzR* mRNA from dissected abdominal fat body (figure 1*e*). With a global *actin*-Gal4 driver (*Act5C*-Gal4), we found a 50% decrease in *CrzR* expression determined from whole flies (electronic supplementary material, figure S1*e*). Knockdown of *CrzR* in the fat body with the *ppl*-Gal4 driver leads to increased Crz peptide levels in the DLPs (figure 1*f–i*) and elevated *Crz* transcript levels in starved flies (figure 1*j*,*k*), presumably as a feedback compensation.

### 2.2. Crz signalling to the periphery modulates stress resistance

**Figure 2.**
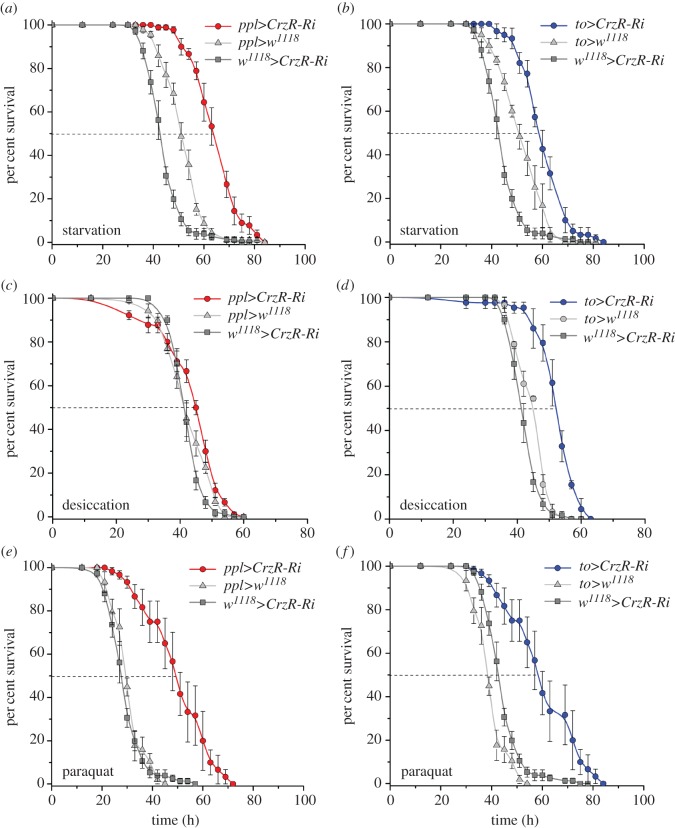
Knockdown of CrzR in the fat body increases stress resistance in flies. We used 3-day-old male flies for all experiments. All results were analysed by log-rank test and data for flies with CrzR-knockdown (*CrzR-Ri*) were compared with their respective controls. Data are presented as means ± s.e.m. (*n* = 80–100 flies for each genotype, run in four replicates). (*a*,*b*) Flies with CrzR knockdown targeted to adipocytes displayed increased survival at starvation (water, but no food; *χ*^2^ = 35.5, *p* < 0.0001 and *χ*^2^ = 22.3, *p* < 0.0001). (*c*,*d*) Knockdown of CrzR with *to*-Gal4 results in enhanced survival under desiccation (no food, no water; *χ*^2^ = 48.6, *p* < 0.0001), whereas the same manipulation using *ppl*-Gal4 does not affect survival (*χ*^2^ = 3.8, *p* = 0.0519). (*e*,*f*) *to*-Gal4-driven *CrzR-*RNAi drastically increased oxidative stress resistance (food supplemented with 10 mM paraquat; *χ*^2^ = 71, *p* < 0.0001) and a similar phenotype was observed with *ppl*-Gal4-driven *CrzR*-RNAi (*χ*^2^ = 11.6, *p* < 0.001).

We previously found that knockdown of *Crz* in DLPs results in increased survival of flies exposed to starvation [32]. Here, we obtained a similar starvation phenotype following *CrzR* knockdown in the fat body using *ppl-*Gal4 and *to*-Gal4 drivers in adult flies (figure 2*a*,*b*). We cannot exclude a direct or indirect effect of the *CrzR*-RNAi on the salivary glands in some of the phenotypes obtained in our assays, and henceforth we refer to our experiments (fat body/salivary gland-specific *CrzR*-knockdown with *to* and *ppl*-Gal4 lines) as targeting *CrzR*-*RNAi* to the periphery. Diminishing the Crz signalling to the periphery by crossing the *ppl-*Gal4 or *to-*Gal4 drivers with UAS-*CrzR-*RNAi results in significantly extended median survival compared with corresponding controls (figure 2*a*,*b*). However, under desiccation conditions (dry starvation), knockdown of CrzR with the *ppl*-Gal4 did not affect fly survival, whereas with the *to*-Gal4 survival increased substantially (figure 2*c*,*d*). The different results here could possibly be explained by the different strength or tissue expression of the two drivers (electronic supplementary material, figure S1). In addition, knockdown of CrzR with both drivers leads to an improved resistance to oxidative stress induced by feeding paraquat-containing food (figure 2*e*,*f*). However, the time of recovery from chill coma (induced by exposure to 0°C for 4 h) was not affected by *CrzR*-*RNAi* in the periphery (electronic supplementary material, figure S2). In summary, resistance to starvation, desiccation and oxidative stress all increased after diminishing Crz signalling to the periphery.

### 2.3. Crz affects carbohydrate metabolism by direct action on peripheral targets, especially during stress

**Figure 3.**
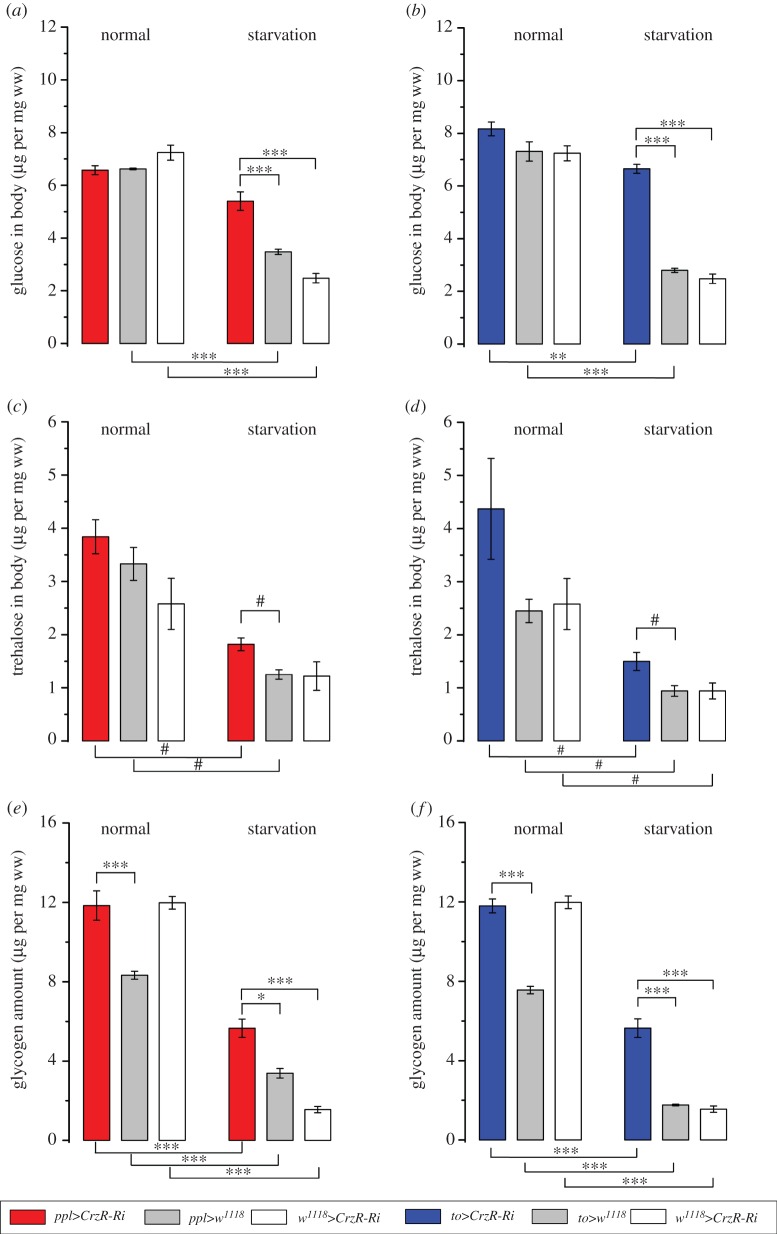
CrzR-knockdown targeted to the periphery increases stored carbohydrates in flies exposed to 36 h of starvation. (*a*,*b*) In flies with CrzR-RNAi targeted to fat body (*ppl*>*CrzR*-*Ri* and *to*>*CrzR*-*Ri*) concentrations of body glucose are about twofold higher than in controls after 36 h starvation, but CrzR-knockdown has no effect in fed flies. (*c*,*d*) CrzR-knockdown in fat body results in higher levels of whole body trehalose after starvation than in control flies, but in fed flies no significant difference is seen. (*e*,*f*) Glycogen stores are depleted by starvation, but flies with *CrzR*-*Ri* targeted to adipocytes contain more glycogen in both experimental conditions used. Data are presented as means ± s.e.m., *n* = 4 replicates with 10–15 flies in every replicate (*p < 0.05, **p < 0.01 and ***p < 0.001; ANOVA followed by Tukey's test, or ^#^*p* < 0.05 (Kruskal–Wallis's test followed by pairwise comparisons using Wilcoxon's rank sum test). See also electronic supplementary material, figure S3 for graphs with circulating carbohydrates and whole body triacylglycerides (TAG).

Similar to mammals, *Drosophila* strictly controls carbohydrate homeostasis. Thus, glucose and trehalose levels in the circulation are tightly regulated by action of DILPs and AKH [42–47]. These fly peptides are therefore the functional homologues of insulin and glucagon. We asked whether the Crz signalling to the periphery also influences carbohydrate metabolism. Previous work showed that Crz knockdown in brain DLPs led to elevated levels of circulating glucose [32]. Here we find that knockdown of CrzR in the fat body does not alter glucose levels in the haemolymph under normal feeding conditions (electronic supplementary material, figure S3*a*). However, in response to starvation, flies with diminished CrzR exhibited higher levels of circulating glucose compared with controls (electronic supplementary material, figure S3*b*). A similar effect was observed on whole-body glucose levels, where diminishing the CrzR in the periphery had no effect on fed flies, but starved flies with reduced receptor expression displayed higher levels of body glucose than controls (figure 3*a*,*b*). Trehalose is the main carbohydrate fuel and a source for both circulating and body glucose in all insects, including *Drosophila* [46,48,49]. Indeed, we found that flies with CrzR-knockdown displayed higher levels of whole-body trehalose following starvation (figure 3*c*,*d*), but circulating trehalose levels were not affected (electronic supplementary material, figure S3*c*,*d*).

A primary carbohydrate store in most animals is glycogen. The major hormone involved in initiating glycogen breakdown in *Drosophila* and other insects is AKH [45,49,50]. A previous study suggests that Crz signalling also affects glycogen storage: flies with Crz knockdown in brain DLPs displayed approximately 1.5 times more glycogen than the controls [32]. That study, however, did not reveal whether the Crz action is via the fat body, or by indirect mechanisms (e.g. via CNS neurons). Here we show that reducing Crz signalling to the fat body/salivary glands by targeted CrzR-knockdown resulted in glycogen stores that were larger than in controls (figure 3*e*,*f*). Moreover, the difference in glycogen stores between control and flies with CrzR-knockdown was more drastic after starvation, where *to*>*CrzR*-RNAi flies contained about three times more glycogen than their respective controls, whereas *ppl*>*CrzR*-*RNAi* flies displayed almost twice as much (figure 3*e*,*f*). Furthermore, 36 h starvation led to a drastic (60–90%) depletion of stored glycogen in control flies, whereas flies with CrzR-knockdown only lost about half of their glycogen (figure 3*e*,*f*). As the steady-state level of glucose in *Drosophila* is known to be supported by glycogen breakdown, trehalose digestion, food ingestion and gluconeogenesis [16,46,51–53], we hypothesize that knockdown of CrzR on peripheral targets might slow down glycogenolysis and stimulate gluconeogenesis and glycogenesis to decrease storage utilization and allow flies to keep high levels of both glucose and glycogen. It can also be noted that flies with diminished Crz signalling (due to genetic deletion or hyperpolarization of Crz neurons) display reduced locomotor activity during starvation [24], which could result in higher levels of carbohydrate.

In contrast with stored carbohydrates, levels of stored lipids, measured as triacylglycerides (TAG), were not affected by knockdown of CrzR in the periphery (electronic supplementary material, figure S3*e*,*f*). However, as expected, the TAG content decreased in all investigated genotypes after starvation compared with fed animals (electronic supplementary material, figure S3*e*,*f*; tables 1 and 2). Finally, in flies with CrzR-knockdown (*to*-Gal4) the body mass was found higher both in fed and starved conditions, compared with the controls (electronic supplementary material, figure S4*a*,*b*; tables 1 and 2).

**Table 1.** Body weight, concentrations of circulating carbohydrate and content of total triacylglycerides (TAG) in control and experimental flies (*ppl-Gal4>CrzR*-RNAi).

genotype	normal (N)	starvation (S)	comparison N versus S
TAG (µg per mg wm)			
*ppl>CrzR-Ri*	3.06 ± 0.15	1.75 ± 0.12	***p < 0.001
*ppl>w^1118^*	3.14 ± 0.24***	1.61 ± 0.06*	***
*w^1118^>CrzR-Ri*	2.66 ± 0.10^n.s.^	0.55 ± 0.05***	***
body mass (mg)			
*ppl>CrzR-Ri*	0.616 ± 0.006	0.537 ± 0.006	***
*ppl>w^1118^*	0.591 ± 0.004^n.s.^	0.554 ± 0.001	*p < 0.05
*w^1118^>CrzR-Ri*	0.640 ± 0.007	0.586 ± 0.007**	**p < 0.01
haemolymph glucose (µmol per mg wm)			
*ppl>CrzR-Ri*	9.68 ± 0.95	11.87 ± 0.57	n.s.
*ppl>w^1118^*	9.44 ± 0.29	8.31 ± 0.42*	n.s.
*w^1118^>CrzR-Ri*	10.15 ± 0.36	5.68 ± 0.42***	**p < 0.01
haemolymph trehalose (µmol per mg wm)			
*ppl>CrzR-Ri*	2.06 ± 0.31	2.55 ± 0.29	n.s.
*ppl>w^1118^*	2.20 ± 0.21	2.26 ± 0.38	n.s.
*w^1118^>CrzR-Ri*	2.31 ± 0.32	2.90 ± 0.44	n.s.

**Table 2.** Body weight, concentrations of circulating carbohydrate and content of total triacylglycerides (TAG) in control and experimental flies (*to-Gal4>CrzR-*RNAi).

genotype	normal (N)	starvation (S)	comparison N versus S
TAG (µg per mg wm)			
*to>CrzR-Ri*	2.49 ± 0.11	0.67 ± 0.05	***p < 0.001
*to>w^1118^*	2.38 ± 0.21***	0.66 ± 0.06***	***
*w^1118^>CrzR-Ri*	2.66 ± 0.10^n.s.^	0.55 ± 0.05***	***
body mass (mg)			
*to>CrzR-Ri*	0.708 ± 0.007	0.643 ± 0.014	***
*to>w^1118^*	0.620 ± 0.005	0.575 ± 0.005***	***
*w^1118^>CrzR-Ri*	0.640 ± 0.007	0.586 ± 0.007***	**p < 0.01
haemolymph glucose (µmol per mg wm)			
*to>CrzR-Ri*	10.67 ± 0.61	13.64 ± 0.45	n.s.
*to>w^1118^*	9.46 ± 1.03	6.27 ± 0.41***	n.s.
*w^1118^>CrzR-Ri*	10.15 ± 0.36	5.68 ± 0.42***	**p < 0.01
haemolymph trehalose (µmol per mg wm)			
*to>CrzR-Ri*	3.49 ± 0.25	2.80 ± 0.36	n.s.
*to>w^1118^*	3.62 ± 0.97	2.57 ± 0.18	n.s.
*w^1118^>CrzR-Ri*	2.31 ± 0.32	2.90 ± 0.44	n.s.

### 2.4. Crz signalling to the periphery affects food intake

**Figure 4.**
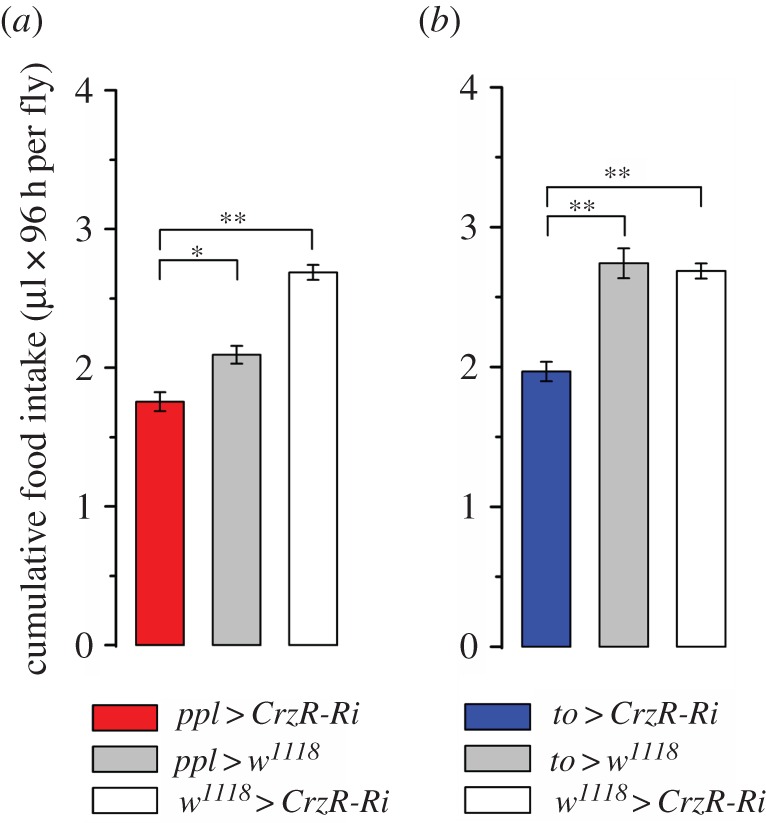
Flies with CrzR-knockdown targeted to adipocytes/salivary glands feed less. Accumulated food intake over 4 days of 3-day-old male flies (µl 96 h per fly) was estimated by CAFE assay. Data are presented as means ± s.e.m., *n* = 3 replicates with 10 flies in each replicate (*p < 0.05, **p < 0.01 from the indicated group as assessed by ANOVA, followed by Tukey's test).

Next, we assessed whether the increased resistance to stress and higher levels of glucose, trehalose and glycogen in flies with CrzR-knockdown could be attributed to differences in food intake prior to exposure to stress. We found that CrzR-knockdown in the periphery led to a significant decrease (15–35%) in cumulative food consumption compared with controls (figure 4*a*,*b*). Temporal dynamics of food intake are shown in electronic supplementary material, figure S4*a*,*b*. The body mass in *to*>*CrzR*-RNAi flies is larger than in controls, both after normal feeding and after starvation (electronic supplementary material, figure S5*a*), whereas no significant difference was seen after *ppl*>*CrzR*-RNAi (electronic supplementary material, figure S5*b*). On the other hand, the stores of carbohydrates were higher in starvation-exposed flies after CrzR-knockdown with both drivers. The finding that the two fat body Gal4 drivers also target the salivary glands (electronic supplementary material, figure S1) suggests that part of the feeding phenotype may derive from alteration of gland function, such as lubrication of the food and secretion of digestive enzymes [54,55]. Nevertheless, our findings suggest that elevated Crz signalling coordinates increased food intake and reallocation of energy stores to alleviate stress and regain metabolic homeostasis.

### 2.5. Diminished CrzR in the periphery influences dilp expression and AKH levels

**Figure 5.**
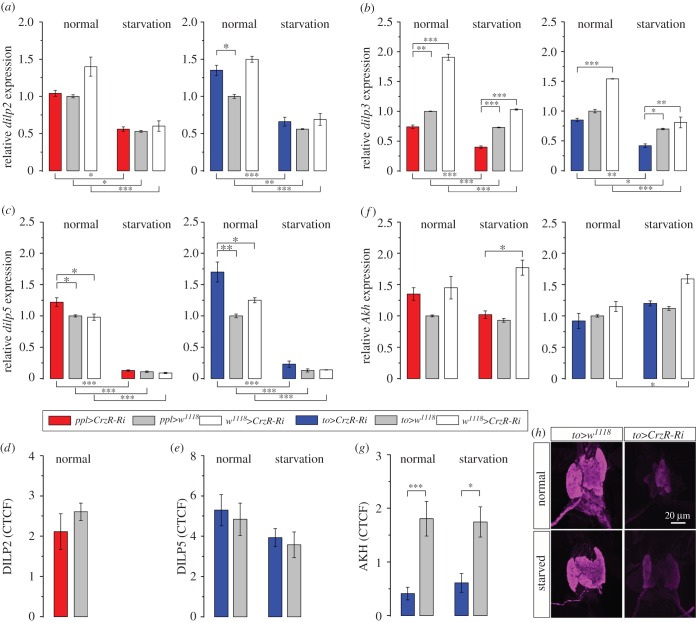
CrzR-knockdown targeted to fat body/salivary gland differentially affects levels of *dilp* transcripts and AKH peptide in fed and starved flies. (*a*) Starvation leads to a strong decrease of *dilp2* mRNA for all genotypes, but is not affected by *CrzR*-RNAi. (*b*) *dilp3* expression is lower in both *ppl*>*CrzR*-Ri and *to*>*CrzR*-Ri flies (compared with controls) in both normal and starvation conditions. (*c*) *dilp5* mRNA expression is very low after starvation, but a higher *dilp5* level in *CrzR*-Ri compared with controls was seen only under normal feeding conditions. (*d*) The DILP2 immunolabelling intensity in IPCs was not affected by CrzR-knockdown in fat body. (*e*) The DILP5 immunolabelling intensity in IPCs was also not affected by *CrzR*-RNAi. (*f*) The *Akh* transcript level was not affected by CrzR-RNAi in fat body with either driver. (*g*,*h*) The AKH peptide level as measured by immunolabelling in corpora cardiaca was drastically reduced in flies with CrzR-knockdown in fat body. In these graphs, data are presented as means ± s.e.m., *n* = 3–4 independent replicates with 10–15 flies in each replicate (*p* < 0.05, **p < 0.01, ***p < 0.001; ANOVA followed with Tukey's test).

Metabolic homeostasis and resource allocation in *Drosophila* is regulated by peptide hormones produced by brain and corpora cardiaca, a functional homologue of the pituitary gland, as well as factors produced by adipocytes of the fat body [16,17,51,56]. Among these are DILPs and AKH [43,44,46,57]. Here we used qPCR to monitor transcripts of *dilps* and *Akh* in flies with CrzR-knockdown in the periphery to determine whether the fat body produces feedback signals to the brain/corpora cardiaca. First, we measured the expression of *dilp2*, *dilp3* and *dilp5*, which are expressed primarily in the insulin-producing cells (IPCs) of the brain [43,58]. The *dilp5* levels were higher in fed flies with diminished CrzR, but not in starved ones (figure 5*c*). However, *dilp2* did not change after CrzR-knockdown, whereas the expression of *dilp3* decreased both in fed and starved flies compared with controls (figure 5*b*) and the *dilp5* expression increased in fed flies (figure 5*c*). The effect of 36 h starvation (compared with fed flies) was consistent for all genotypes: we noted a significant decrease in mRNA levels of *dilp2*, *dilp*3 and *dilp5* (figure 5*a–c*). Immunolabelling with antisera to DILP2 and 5 did not reveal any significant effect of CrzR-knockdown on peptide levels in IPCs (figure 5*d*,*e*), perhaps suggesting that peptide release was not affected [59]. Neither manipulation of CrzR nor starvation affected expression of *Akh* mRNA (figure 5*f*), but AKH immunolabelling in the corpora cardiaca decreased drastically after CrzR-knockdown, both in fed and starved flies (figure 5*g*,*h*). Reduced AKH immunolabelling in the corpora cardiaca may reflect an increased release of this peptide.

### 2.6. Knockdown of CrzR in the periphery affects expression of genes regulating metabolism and stress

**Figure 6.**
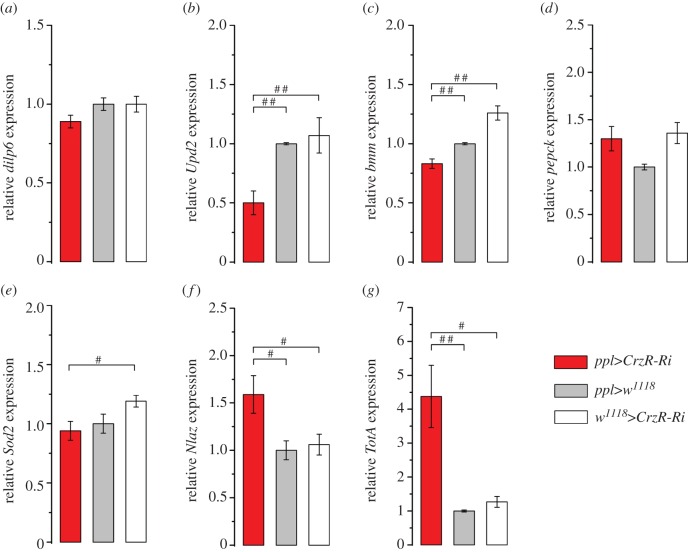
Changes in expression of gene transcript in dissected fat body determined by qPCR in *ppl>CrzR*-RNAi flies. (*a*) The *dilp6* transcript is not affected in *ppl>CrzR-Ri* flies. (*b*) The cytokine unpaired-2 (*Upd2*) mRNA is reduced in the fat body. (*c*) The brummer TAG lipase (encoded by *bmm*) transcript is decreased. (*d*) Expression of the transcript of phosphoenolpyruvate carboxykinase (encoded by *pepck*) is not affected. (*e*) No change was detected in mRNA of *Superoxide dismutase 2* (*Sod2*). (*f*) The stress responsive gene *Neural Lazarillo* (*Nlaz*) increased after CrzR-RNAi. (*g*) Another stress inducible gene, *Turandot A* (*TotA*), was strongly activated in fat body of flies with *CrzR*-RNAi. The expression levels in dissected abdominal fat body were calculated with the 2^−ΔΔCt^ method relative to the control *ppl>w^1118^*, which is set at one. Data are presented as means ± s.e.m., *n* = 3 independent replicates with 20–30 fat body samples in each replicate Kruskal–Wallis's test followed by pairwise comparisons using Wilcoxon's rank sum test with (^#^*p* < 0.05, ^##^*p* < 0.01).

In response to changing levels of circulating carbohydrates, lipids or amino acids, several factors are known to be secreted from the nutrient-sensing fat body and act on neurosecretory cells of the brain, which in turn signal to the fat body and other tissues to affect metabolism and nutrient stores [37,60–63]. One fat-body-derived coordinator of metabolism and growth is DILP6 [64,65]. We found that knockdown of CrzR (with *ppl*-Gal4) did not affect *dilp6* mRNA expression in dissected abdominal fat body (figure 6*a*). Also, in whole-fly extract we did not find any differences in *dilp6* expression after CrzR-knockdown compared with controls, or between fed and starved flies (electronic supplementary material, figure S6*a*). Another factor secreted from the *Drosophila* fat body and acting on the brain IPCs is the cytokine Upd2 (unpaired-2) with leptin-like properties [37]. The *Upd2* transcript was decreased in fat body extract after CrzR-knockdown (figure 6*b*), but was not significantly altered in whole fly extracts (electronic supplementary material, figure S6*b*). Possibly the altered *Upd2* expression in the fat body reflects altered Upd2 signalling to the brain as a consequence of *CrzR*-RNAi in the fat body.

Next, we measured transcript of the gene *bmm*, encoding Brummer TAG lipase, a protein responsible for lipid breakdown [66]. *bmm* levels diminished in fat body extract after CrzR-knockdown (figure 6*c*), but increased slightly in the whole fly after *to*>*CrzR*-RNAi (electronic supplementary material, figure S6*c*). In all genotypes, *bmm* expression was strongly increased after starvation compared with fed flies (electronic supplementary material, figure S6*c*). In fat body extract *CrzR*-RNAi had no effect on *phosphoenolpyruvate carboxykinase* (*pepck*), a gene encoding a key enzyme in gluconeogenesis in both *Drosophila* and mammals [67] (figure 6*d*). Also, in whole fly extracts *CrzR*-*RNAi* had no effect, although starvation induced an increase of *pepck* in all flies (electronic supplementary material, figure S6*d*).

**Figure 7.**
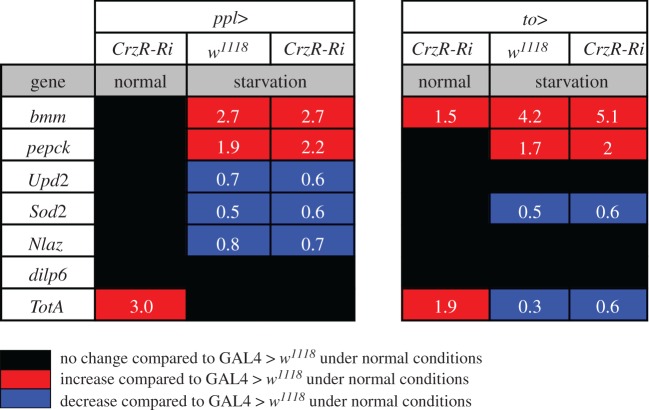
Summary of changes in gene transcripts measured from whole flies after *CrzR*-RNAi with two different fat body Gal4 drivers. Knockdown of the CrzR in fat body does not induce a strong response in gene transcription at the level of whole flies during normal (fed) conditions. Data from qPCR are shown as average fold-changes of expression compared with level in the respective controls during normal conditions (see key for colour coding). Both experimental fly crosses, *ppl*>*CrzR*-*Ri* and *to*>*CrzR*-*Ri*, display similar patterns of gene transcript changes. Thus, *TotA* was upregulated during normal conditions in flies with CrzR-knockdown, but starvation results in reduced *TotA*. *dilp6* mRNA is not affected in whole flies by any condition. Starvation leads to downregulation of *Upd2*, *Sod2* and *Nlaz*, whereas *bmm* and *pepck* expression increases. In electronic supplementary material, figure S6, these experiments are shown in regular graphs.

To determine whether Crz signalling to the periphery plays a role in general stress response, we monitored the expression of genes encoding superoxide dismutase 2 (*Sod2*) [68], Neuronal Lazarillo (*NLaz*) as a target of JNK signalling [13] and Turandot A (*TotA*) as a read-out gene in JAK/STAT signalling [69,70] in whole flies, as well as in dissected abdominal fat body. *Sod2* was not altered by *CrzR*-RNAi (figure 6*e*), but displayed higher expression in fed flies than in starved ones (electronic supplementary material, figure S6*e*). In the fat body samples *NLaz* was upregulated after *CrzR*-RNAi (figure 6*f*), but no difference was detected in whole body extract (electronic supplementary material, figure S6*f*). *TotA* expression was upregulated in response to *CrzR*-RNAi, and this was seen both in whole body and fat body extracts (figure 6*g*; electronic supplementary material, figure S4*g*). *TotA* was also found higher in fed flies compared with starved ones. Thus, we obtained some evidence for effects of CrzR-knockdown in the periphery on genes involved in general stress responses. A summary of results for whole-body measurements is shown in figure 7.

### 2.7. Knockdown of the CrzR with a CrzR-Gal4 affects stress response and food intake

**Figure 8.**
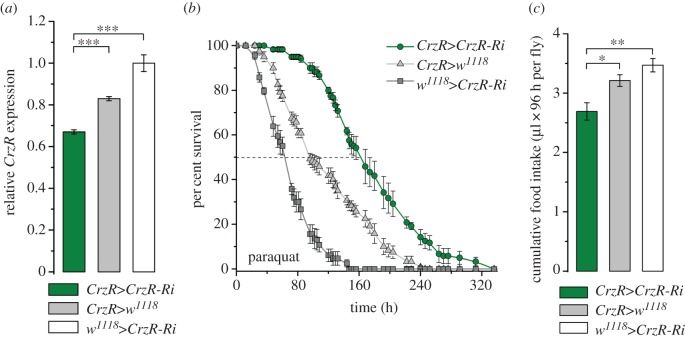
CrzR-knockdown with a *CrzR*-Gal4 driver generates phenotypes similar to peripheral receptor knockdown. (*a*) Knockdown efficiency of *CrzR*-RNAi with the *CrzR*-Gal4. Data are presented as means ± s.e.m., *n* = 4 independent replicates with 10–15 flies in each replicate (***p < 0.001, ANOVA followed with Tukey's test). (*b*) Resistance to oxidative stress induced by paraquat feeding increases after *CrzR*-RNAi (*χ*^2^ = 137 and 375, *p* < 0.0001 comparing with the Gal4 and UAS control, respectively). (*c*) Food intake is reduced after *CrzR*-RNAi. Accumulated food intake over 4 days of 3-day-old male flies (µl 96 h per fly) was estimated by CAFE assay. Data are presented as means ± s.e.m., *n* = 4 independent replicates with eight flies in each replicate (*p* < 0.05, **p < 0.01, ANOVA followed with Tukey's test).

We used a *CrzR*-Gal4 to diminish the CrzR more broadly as a comparison with our experiments on fat body (peripheral) directed RNAi. It is known that the CrzR in *Drosophila* is expressed also in neurons of the CNS [35,39], salivary glands and the dorsal vessel, also known as heart (see FlyAtlas) [36]. Nevertheless, we found that *CrzR*-Gal4>UAS-*CrzR*-RNAi resulted in a similar increased resistance to paraquat-induced oxidative stress and a decrease in food intake (figure 8) as seen after targeting *CrzR*-RNAi to the periphery. Thus, the additional neuronal knockdown of the receptor did not seem to affect the outcome of the assays tested.

### 2.8. Knockdown of Crz peptide with a Crz-Gal4 affects stress responses and food intake

**Figure 9.**
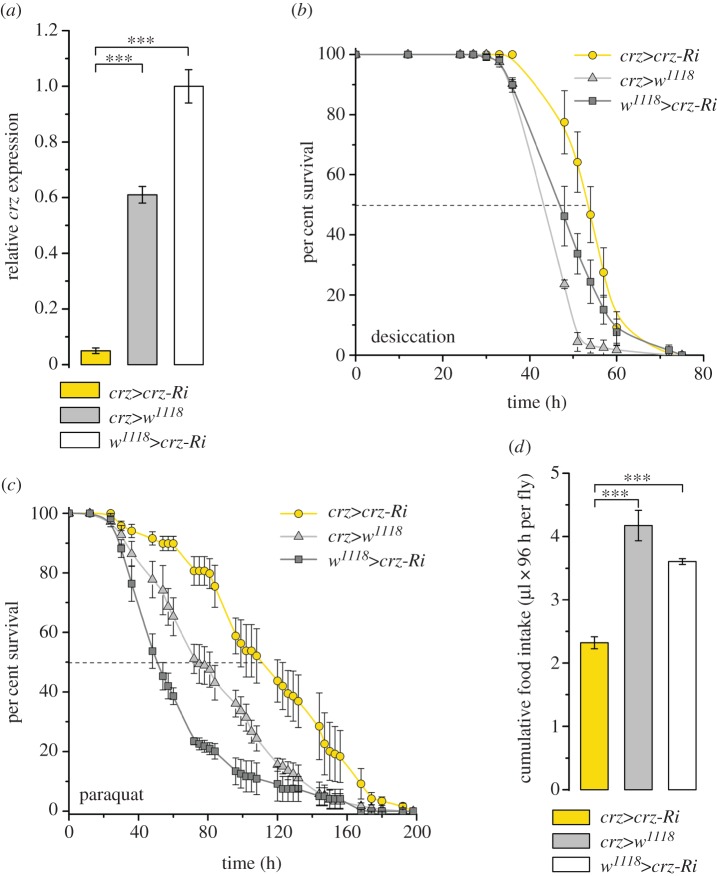
Crz knockdown with a *Crz*-Gal4 driver generates phenotypes similar to peripheral receptor knockdown. (*a*) Knockdown efficiency of *Crz*-RNAi with the *Crz*-Gal4. Data are presented as means ± s.e.m., *n* = 4 independent replicates with 10 flies in each replicate (***p < 0.001, ANOVA followed with Tukey's test). (*b*) Resistance to desiccation stress is increased after *Crz*-RNAi. (*c*) Resistance to oxidative stress induced by paraquat feeding increases after *Crz*-RNAi (*χ*^2^ = 54 and 137, *p* < 0.0001 comparing with the Gal4 and UAS control, respectively). (*d*) Food intake is reduced after *Crz*-RNAi. Accumulated food intake over 4 days of 3-day-old male flies (µl 96 h per fly) was estimated by CAFE assay (*n* = 4 independent replicates with eight flies in each replicate; ***p < 0.001, ANOVA followed with Tukey's test).

In an earlier study, we showed that diminishing Crz peptide levels broadly by *Crz*-Gal4>UAS-*Crz*-RNAi increased starvation resistance in fed flies [32]. Here, we extended these findings by showing that *Crz*-Gal4>UAS-*Crz*-RNAi increases survival both during desiccation and paraquat feeding, as well as diminishes food intake (figure 9). Taken together, we therefore show that global knockdown of Crz peptide or CrzR yields effects on stress resistance and feeding very similar to those noted after CrzR-knockdown in the periphery.

## 3. Discussion

Our study shows that the CrzR, the insect homologue of GnRH receptors, is expressed in the fat body and salivary glands of adult flies. The fat body thus receives Crz signals from the brain that affects carbohydrate but not lipid metabolism, diminishes resistance to starvation, desiccation and oxidative stress, increases food ingestion, and triggers feedback signals from the fat body to the brain. We cannot exclude the possibility that part of these phenotypes are caused by Crz signalling to the salivary gland. However, our aim was to tease apart the effects of systemic Crz signalling from Crz action in the CNS. Several of the effects of diminishing the CrzR in the periphery are more prominent in starved flies. Thus, we suggest that Crz signalling from neuroendocrine cells of the brain to the fat body (and perhaps salivary glands) is important when flies are under nutritional stress. In mammals, the GnRH producing neuroendocrine cells are located in the hypothalamus and receive nutrient inputs via leptin signalling [30,31], and the GnRH system is also affected by stress [9,10]. The *Drosophila* neuroendocrine cells releasing Crz are located in a brain area that is a functional equivalent of the hypothalamus [71], and may be regulated by nutrient-sensing inputs [60], and stress hormones such as the diuretic hormones DH_31_ and DH_44_ that are ancestrally related to calcitonin and corticotropin-releasing factor, respectively [23,25].

It has previously been suggested that Crz is utilized in stress signalling in various insects [22,23], but only a few studies in *Drosophila* have actually tested this. An earlier report showed that ablation or inactivation of the Crz-producing DLP neurons in the brain resulted in flies with increased resistance to metabolic, oxidative and desiccation stress, as measured by survival, and also resulted in increased triglyceride levels [24]. The same authors also found that the *Crz* transcript decreased during starvation and osmotic, but not oxidative stress. Furthermore, ablation of Crz neurons resulted in elevated dopamine levels in the circulation and increased locomotor activity in male flies [24]. However, these results need to be interpreted with caution as a later study found that another neuropeptide, short neuropeptide F (sNPF), coexpressed with Crz in DLPs, also affects starvation resistance and other metabolism related phenotypes [32]. Nonetheless, our previous paper demonstrated that knockdown of Crz in DLPs in the *Drosophila* brain increased starvation resistance and carbohydrate and TAG levels [32].

Our results herein are derived from selectively diminishing Crz signalling to the fat body and salivary glands by targeted CrzR-knockdown. Thus, we can discount direct actions on other targets, including the brain and heart. Nevertheless, the effects seen here following CrzR-knockdown in the periphery on stress resistance and carbohydrate metabolism are similar to those where DLP neurons were targeted by Crz-*RNAi* [24,32]. We also showed here that more global knockdown of CrzR or Crz peptide resulted in stress and feeding phenotypes very similar to those obtained after more selective CrzR-*RNAi* in fat body/salivary gland. Therefore, it seems that a substantial portion of the systemic effects of Crz are mediated via the fat body. Indeed, we found clear effects of CrzR-knockdown on transcription of a few relevant genes in the fat body (figures 6 and 7). The *bmm* transcript level decreased*. bmm* encodes the TAG lipase Brummer, which regulates lipid storage [66], and this gene is therefore important in regulation of energy homeostasis. However, as will be discussed in more detail below, we also detected effects of CrzR-knockdown on the fat body genes *Upd2*, *NLaz* and *TotA.* Upd2 is a leptin-like factor, which is nutrient signal released from the fat body acting on the brain IPCs [37]. Such, a feedback to the brain is supported by changes in transcripts of *dilp3* and *5*, as well as AKH peptide levels after CrzR-knockdown. This feedback may thus result in complex effects after CrzR-knockdown in the fat body due to both direct and indirect regulation of the adipocytes by Crz as well as DILPs and AKH.

Earlier studies have shown that Crz displays multiple actions in insects, several of which may be associated with stress responses. Crz was first identified as a cardioactive hormone in cockroaches [72], but its actions have been extended in *Drosophila* to roles in reproduction [39,73], carbohydrate metabolism [24,38], modulation of locomotor activity [24], regulation of ethanol sedation and metabolism [26,35], and a role in the clock system [34,74]. In other insects, Crz induces gregarization-associated colour change in locusts [75] and controls ecdysis behaviour in a moth [76]. Furthermore, it was shown recently that during adult reproductive diapause in *Drosophila*, when stress resistance is increased [14,77], transcripts of both Crz and its receptor are significantly upregulated [78]. In addition, a possible function of Crz signalling to the salivary glands remains to be determined. Data from FlyAtlas [36] suggest the presence of the CrzR in the *Drosophila* salivary glands, and we cannot exclude that our *CrzR*-*RNAi* experiments with *ppl*- and *to*-Gal4 drivers generated effects on salivary gland function that contributed to the phenotypes we recorded. Adult salivary gland function is not well investigated in *Drosophila*, but this tissue may contribute to facilitating food ingestion and processing by lubrication and release of digestive enzymes (see [54,55]).

The *Drosophila* Crz receptor is ancestrally related to the GnRH receptor family [79,80], which is known to participate in stress responses in mammals [81,82]. Also, the CrzR and AKH receptor (AkhR) have been proposed to have a common ancestor [79], suggesting that Crz and AKH signalling might share some of the ancient functions in regulation of stress and metabolism. AKH predominantly stimulates catabolic processes (mobilization of lipids, carbohydrates and amino acids) while simultaneously inhibiting their biosynthesis [18,47–49]. Although both AKH and Crz target the fat body, a comparison of our results and those of earlier studies analysing *Akh* and *AkhR* mutants (see electronic supplementary material, figure S7) reveals that these two signalling systems play distinct roles in metabolism and stress responses [18,45,83]. Knockdown of Crz or ablation of Crz-producing cells leads to increased levels of stored lipids and carbohydrates [24,32], and here we show that the effect on carbohydrate metabolism is mediated by Crz signalling to the periphery, and this effect is stronger during stress conditions. One difference between Crz and CrzR-knockdown is the lack of effect on TAG levels after CrzR-knockdown in the periphery. This suggests that Crz regulates lipid metabolism indirectly via another signalling system.

The fat body is not only a primary metabolic tissue and energy store, but it is also an active endocrine organ [2,16,84,85]. Hence, similar to mammals, *Drosophila* displays reciprocal humoral interactions between adipocytes/liver and brain neuroendocrine cells. The adult fat body can release hormonal factors to modulate IPCs and systemic insulin signalling, which in turn signals to the fat body [17,60,63,86]. In mammals, systemic insulin signalling is influenced by adipocyte-derived hormonal factors, such as leptin and adiponectin [87,88]. In *Drosophila*, a functional leptin homologue, Upd2, produced in the fat body, was shown to regulate IPCs [37]. In addition, DILP6 from the fat body can act on IPCs to decrease DILP2 expression [63]. Fasting induces *dilp6* mRNA expression in fat body of *Drosophila* larvae [64] and adults [63]. In our study, 36 h of starvation failed to affect *dilp6* expression but diminished *Upd2* in the fat body. Taken together with the altered *dilp3* and *5* transcript levels, our results suggest that fat-body-derived humoral signals are affected by Crz activation of the adipocytes.

We also assayed a few fat body genes associated with stress signalling in *Drosophila*. Of these, the mRNA of Turandot A (*TotA*) was upregulated after CrzR-RNAi in the periphery. *TotA* is a target gene of Janus kinase/signal transducer and activator of transcription (JAK/STAT) signalling [89], and is known to play an important role in stress tolerance and immune response [69,70,90]. In fed flies, CrzR-knockdown had no effect on the transcript of the antioxidant enzyme manganese-containing superoxide dismutase 2 (*Sod2*) which is a target of the transcription factor FOXO [91,92], but upregulated the lipocalin Neural Lazarillo (encoded by *NLaz*), which is related to apolipoprotein A (ApoA) in mammals and is part of the stress responsive Jun-N-terminal Kinase (JNK) signalling pathway [13,93]. Thus, we can conclude that increased Crz action on the fat body upregulates *TotA* and *Nlaz* stress signalling.

In summary, signalling through the CrzR in the periphery during metabolic stress results in increased nutrient intake and reallocated energy stores enabling the fly to reestablish homeostasis. The Crz action triggers transcriptional changes in the adipocytes that include stress genes and one gene involved in metabolism. The alteration of transcript levels of *Crz*, *dilp3* and *dilp5* and the decreased AKH peptide levels suggests that Crz signalling to the periphery generates a feedback signal to the brain and corpora cardiaca endocrine cells and thereby gives rise to complex hormonal fine-tuning. The CrzR is considered ancestrally related to the GnRH receptor [79], which is known to be involved in specific stress responses in mammals [9,81,82]. Thus, the role of Crz in stress may be an ancient one, and over evolution Crz, AKH and GnRH signalling systems have acquired additional functions seen both in *Drosophila*, other invertebrates and in mammals.

## 4. Material and methods

### 4.1. Fly strains and husbandry

The following *Drosophila melanogaster* Gal4 lines were used: *ppl-*Gal4 [40] from M. Pankratz (Bonn, Germany); *to-Gal4* [41] provided by B. Dauwalder (Houston, TX, USA); Crz-Gal4 [94], *CrzR-Gal4^T11a^* and *CrzR-Gal4^Se^* [35] were gifts from J.H. Park (Knoxville, TN, USA). For targeted interference we used *UAS-Crz-*RNAi (ID:106876) and *UAS-CrzR-*RNAi (ID:44310) from the Vienna Drosophila Resource Center (VDRC). *Act5C*-Gal4 and *w^1118^* were from Bloomington Drosophila Stock Center (BDSC), Bloomington, IN, USA. The JFRC81-10XUAS-Syn21-IVS-GFP-p10 line [95] was obtained from M. Texada (Janelia Farm, Ashburn, VA, USA). This line is referred to as UAS-GFP in the text. Parental flies were reared on BDSC food medium (see http://flystocks.bio.indiana.edu/Fly_Work/media-recipes/bloomfood.htm) supplemented with 1.5 g l^−1^ nipagin. For collection of eggs 4–5-day-old flies were transferred to enriched medium containing 100 g l^−1^ sucrose, 50 g l^−1^ yeast, 12 g l^−1^ agar, 3 ml l^−1^ propionic acid and 3 g l^−1^ nipagin. Experimental flies were grown under uncrowded conditions at 25°C and normal photoperiod (12 L : 12 D).

### 4.2. Immunocytochemistry and imaging

Adult *Drosophila* tissues were dissected in phosphate buffered saline (PBS) (pH 7.2) and fixed in ice-cold 5% paraformaldehyde in 0.1 M sodium phosphate buffer for 3.5 h. Samples were washed thoroughly in PBS before application of primary antisera. The primary antisera were diluted in PBS with 0.5% Triton X (PBST) and tissues were incubated for 48 h at 4°C with gentle agitation.

The following primary antisera were used: rabbit anti-Crz [96] at a dilution of 1 : 4000 rabbit anti-DILP2 [97] at dilution of 1 : 2000 (both were kindly donated by J. A. Veenstra, Bordeaux, France). A rabbit antiserum to a mosquito AKH was donated by M. Brown (Athens, GA). A rabbit anti-DILP5 [98] was applied at a dilution of 1 : 2000 and mouse anti-GFP at a dilution of 1 : 1000. For detection of primary antisera we used Alexa 546 tagged goat anti-rabbit antiserum and Alexa 488 tagged goat anti-mouse antiserum (Invitrogen) at a dilution of 1 : 1000. After washes, tissues were mounted in 80% glycerol in PBS. For each experiment we analysed at least 12 tissues from three independent replicates.

Tissue samples were imaged with a Zeiss LSM 510 META confocal microscope (Jena, Germany) using 40× or 63× oil immersion objectives. Confocal images were processed with Zeiss LSM software and FIJI immunofluorescence levels were recalculated to corrected total cell fluorescence (CTCF) according to Burgess *et al.* [99] using Fiji software [100].

### 4.3. Stress resistance experiments

We used 3-day-old male flies to assay survival under stress conditions. In each replicate, 15 male flies (unless otherwise stated) were kept in 50 ml vials and mortality was monitored every 3 h until no alive flies were left. For starvation resistance flies were placed in vials, containing 5 ml of 0.5% aqueous agarose (A2929, Sigma-Aldrich). For desiccation flies were kept in empty vials without access to water or food. To induce oxidative stress flies were kept on 5 ml of enriched food medium, supplemented with 10 mM paraquat (methyl viologen, 856177, Sigma-Aldrich). For chill coma recovery assay flies were placed into empty vials (15 flies per vial) and vials were placed into ice to induce immediate chill coma. Flies were incubated at 0°C for 4 h followed by a transfer flies to 25°C for recovery. Recovering flies were monitored every 2 min till all flies recovered. All stress resistance assays were done at 25°C and 12 L : 12 D. All experiments were run in three replicates with at least 100 flies of each genotype in each run.

### 4.4. Carbohydrate and lipid assays

Flies from all investigated genotypes were also used to measure concentrations of circulating (haemolymph) glucose, together with stored (whole body) glucose, trehalose and glycogen as well as stored triacylglycerides (TAG). For this we used 10–15 male flies from each of four independent replicates. Three-day-old male flies of each genotype and replicate were separated into two different treatments. One batch of the flies was transferred to 5% agarose for starvation for 36 h (this is the time when control and knockdown flies start to show different survival). Another batch of flies was kept at normal (fed) conditions on the enriched food medium. After 36 h of incubation all flies were frozen in liquid nitrogen and kept for further assays.

Haemolymph was collected following published protocols [101,102] including centrifugation (3000*g*, at 4°C, for 6 min). After haemolymph extraction the pelleted fly bodies were homogenized in 0.01 M PBS buffer (pH 7.2) in ratio 1 : 10 (w/v) followed by centrifugation (16 000*g*, 4°C, 15 min) and collection of supernatants. Carbohydrate assays of haemolymph and whole body supernatants were performed as described earlier [32,77] using a glucose assay kit with glucose oxidase and peroxidase (Liquick Cor-Glucose diagnostic kit, Cormay, Poland). Glucose was measured after trehalose and glycogen from haemolymph and whole-body supernatants had been hydrolysed into glucose with porcine kidney trehalase (Sigma, T8778) and amyloglucosidase from *Aspergillus niger* (Sigma, 10115).

For extraction of triacylglycerides (TAG) 10–15 pre-weighed flies per sample were homogenized in the ratio 1 : 10 (w/v) in PBS buffer (pH 7.4), supplemented with 0.05% Triton X [103]. Homogenates were incubated for 15 min at 100°C, cooled on ice and centrifuged (16 000*g*, 4°C, 10 min). Supernatants were collected and the amount of TAG was determined with a Liquick Cor-TG diagnostic kit (Cormay, Poland) using a linear regression coefficient from a standard curve made with 1.1–22 mg of TAG standard (Cormay, Poland). Absorbance of samples was measured at 550 nm with a spectrophotometer (Genova Jenway, UK-PRC).

### 4.5. Capillary feeding assay

The capillary feeding (CAFE) assay was performed on 3-day-old male flies according to Ja *et al.* [104] with 5 µl capillaries filled with food composed of 50 g l^−1^ sucrose, 50 g l^−1^ yeast and 3 ml l^−1^ propionic acid. Measurements were made in three independent replicates with 10 flies in each replicate. The food consumption was recorded every 24 h with refilling of capillaries. Data are shown as total food intake in microliters of consumed food by one fly for 96 h (µl per 96 h per fly).

### 4.6. Quantitative real-time PCR

Total RNA was extracted with Trizol-chloroform from four independent biological replicates with 10–15 whole flies in each replicate, from both normal (fed) and starvation conditions. We also analysed 20–30 dissected abdomens (carcasses) of normally fed flies with attached fat body. The dorsal part of the carcass was removed to ensure minimal contamination from dorsal vessel, known to express CrzR (see FlyAtlas). Extracted RNA was further treated with DNAase (EN0521, Thermo Fisher Scientific). Quality and concentration of the RNA were determined with a NanoDrop 8000 spectrophotometer (Thermo Fisher Scientific). Reverse transcription (cDNA synthesis) was done following [105] in 20 µl reaction mixture, containing 2 µg of total RNA, 1 µl of 10 mM dNTPs (R0192, Thermo Fisher Scientific), 0.4 µl of 100 µM random hexamer primer (SO142, Thermo Fisher Scientific) and 2 µl of M-MuLV reversible transcriptase (EP0352, Thermo Fisher Scientific) were used. The cDNA was then applied for qPCR using a StepOnePlus (Applied Biosystems) instrument and SensiFAST SYBR Hi-ROX Kit (Bioline) as recommended by the manufacturer. For each sample duplicate reactions of the total volume of 20 µl were conducted with a primer concentration of 400 nM and 4 µl of diluted 1 : 10 cDNA template. The mRNA levels were normalized to that of the reference genes, *rp49* and *Act88* levels in the same samples (displayed data are shown relative to rp49). Relative expression values were determined by the 2^−ΔΔCt^ method [106]. The sequences of the primers are shown in electronic supplementary material, table S1.

### 4.7. Data analysis

The experimental data are presented as means ± s.e.m. Statistical analysis was performed using R statistical software (Foundation for Statistical Computing, Vienna, Austria) v. 3.0.3. Prior to statistical treatment all data were tested for homogeneity of variances using the Fligner–Killeen's test and for normal distribution by the Shapiro-Wilk's normality test. Unless otherwise stated statistical analysis was performed by one-way analysis of variance (ANOVA) followed by Tukey's multiple comparisons test, when data had a normal distribution, and a non-parametric Kruskal–Wallis's test followed by pairwise comparisons using Wilcoxon's rank sum test when data lacked normal distribution. Corrected total cell fluorescence data and qPCR results in dissected fat body samples were compared with unpaired *t*-test. Stress survival data were compared using log-rank test, Mantel-Cox. A 95% confidence limit (*p* < 0.05) was used throughout the study. Graphs were produced in OriginPro v. 7.5 software.

## Supplementary Material

The ppl and to-Gal4 drivers direct GFP expression to abdominal fat body, but also other tissues

## Supplementary Material

Knockdown of CrzR in fat body does not affect recovery from chill coma

## Supplementary Material

Knockdown of CrzR in fat body has minor and variable effects on circulating carbohydrates and total triacylglycerides (TAG)

## Supplementary Material

Diminishing Crz signaling decreases food ingestion

## Supplementary Material

Knockdown of CrzR in fat body/salivary glands results affects body mass

## Supplementary Material

Supplementary Figure 6

## Supplementary Material

Supplementary Figure 7

## Supplementary Material

Table S1
