# Supplementary figures and images for: Systemic corazonin signalling modulates stress responses and metabolism in Drosophila
Source: Open Biol. 2016 Nov 3;6(11):160152. doi: 10.1098/rsob.160152 (PMC5133436; doi:10.1098/rsob.160152)

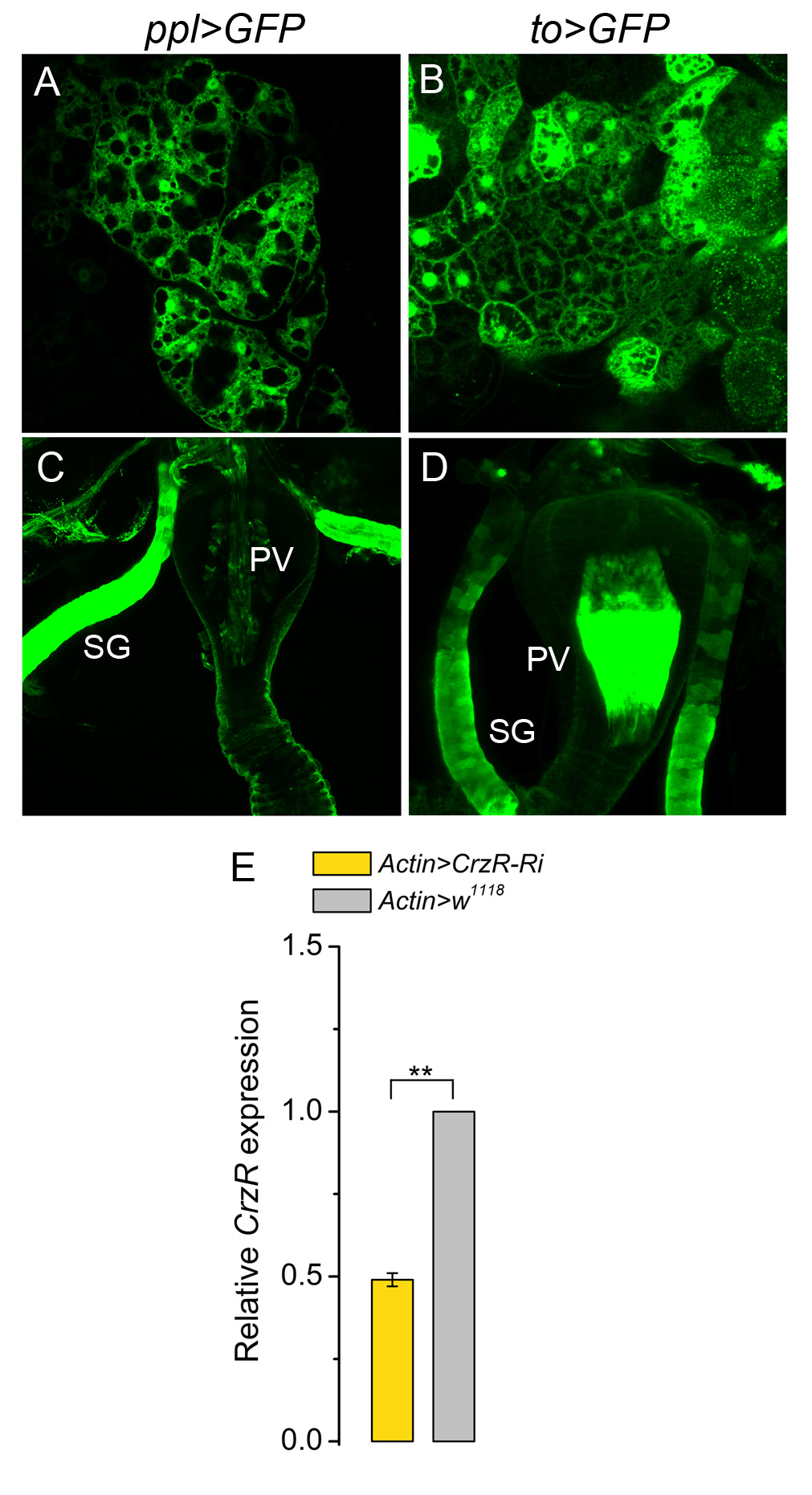

Supplement: The ppl and to-Gal4 drivers direct GFP expression to abdominal fat body, but also other tissues [file rsob160152supp1.jpg]

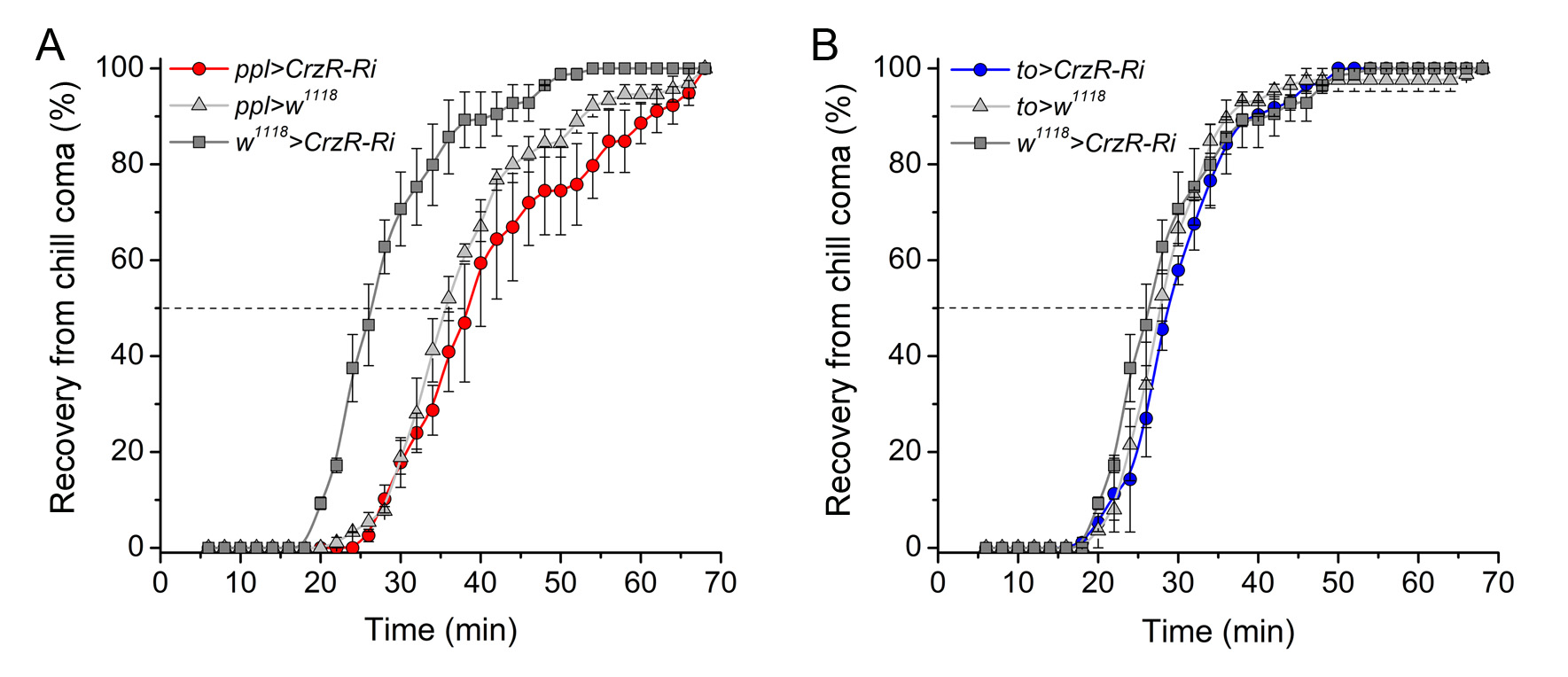

Supplement: Knockdown of CrzR in fat body does not affect recovery from chill coma [file rsob160152supp2.jpg]

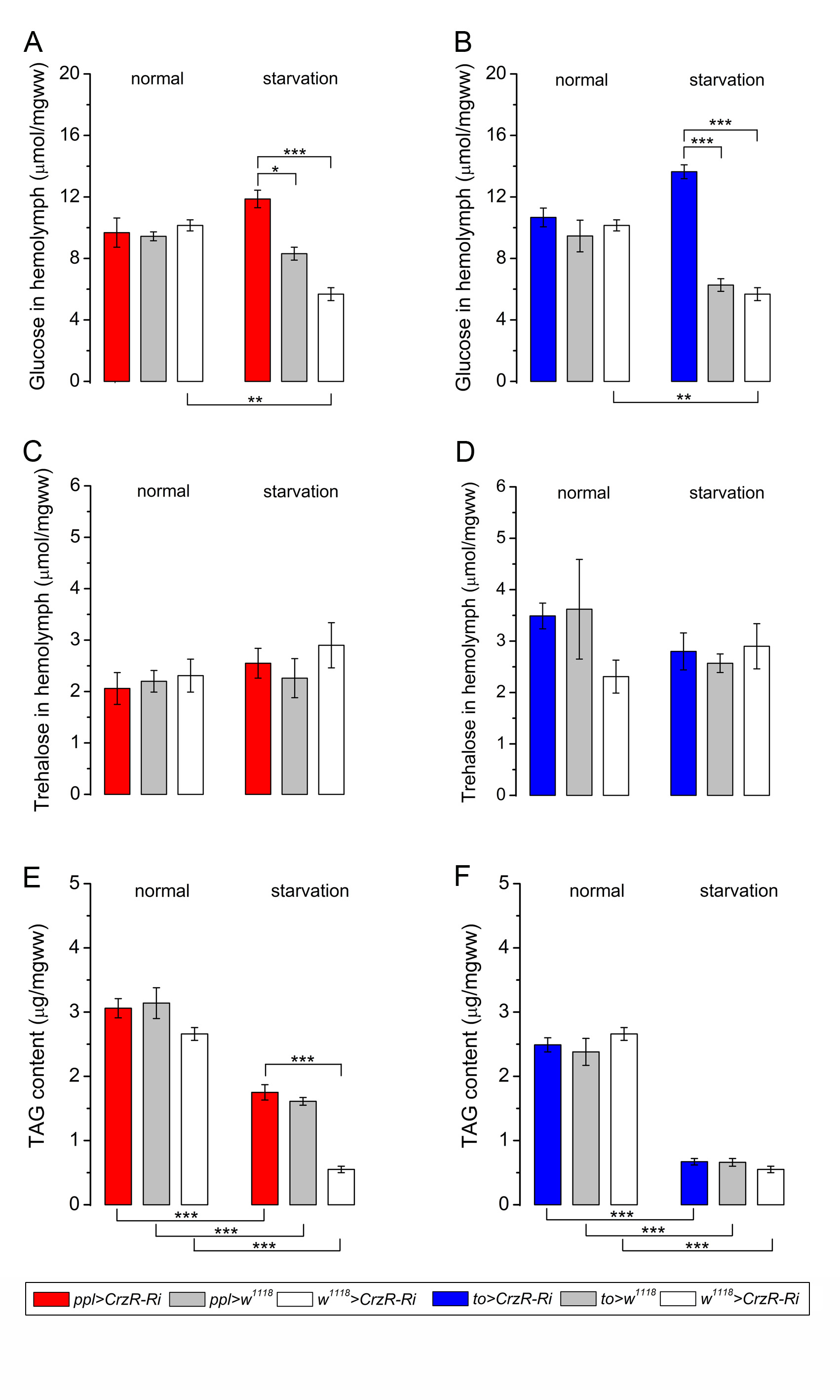

Supplement: Knockdown of CrzR in fat body has minor and variable effects on circulating carbohydrates and total triacylglycerides (TAG) [file rsob160152supp3.jpg]

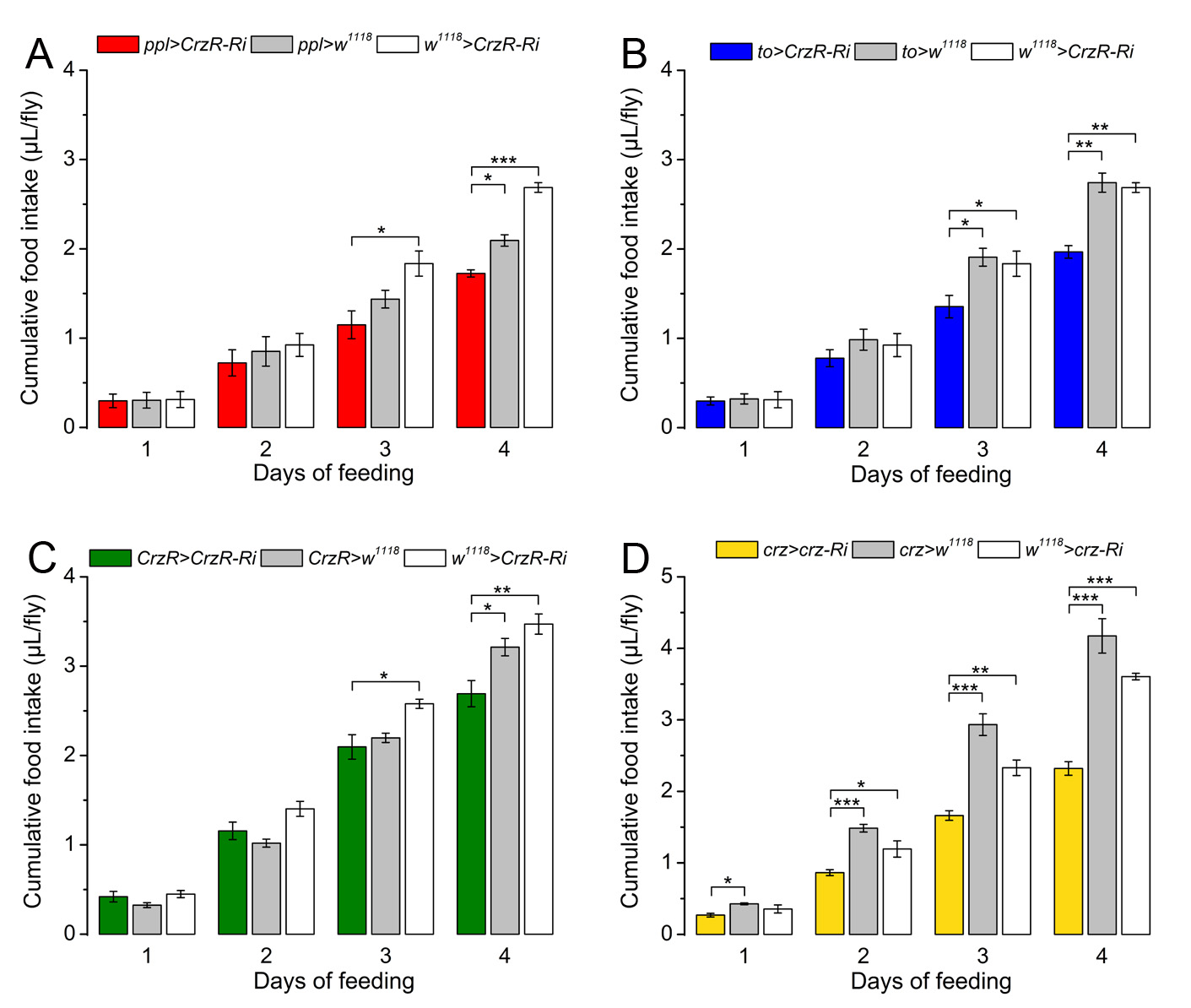

Supplement: Diminishing Crz signaling decreases food ingestion [file rsob160152supp4.jpg]

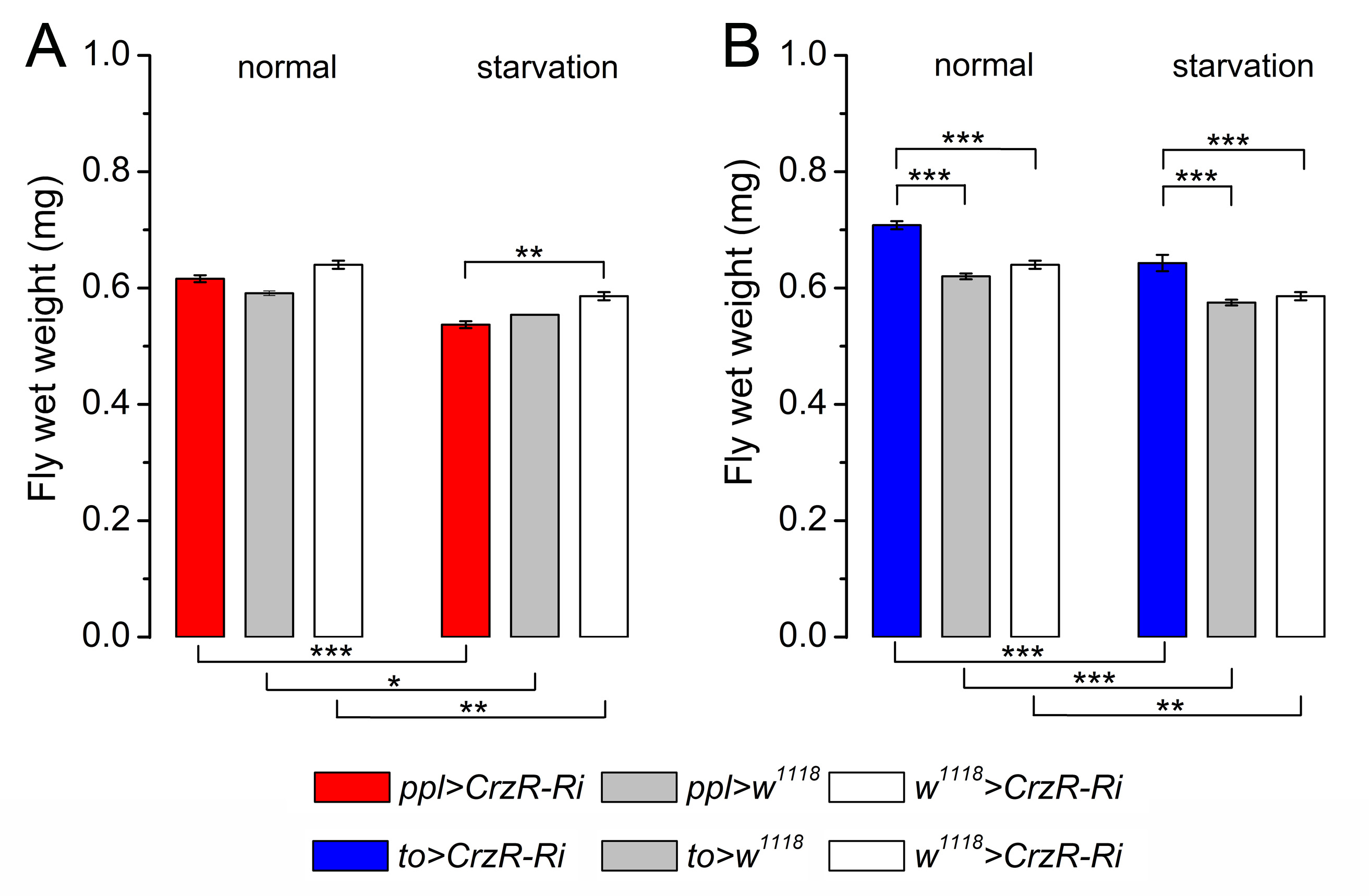

Supplement: Knockdown of CrzR in fat body/salivary glands results affects body mass [file rsob160152supp5.jpg]

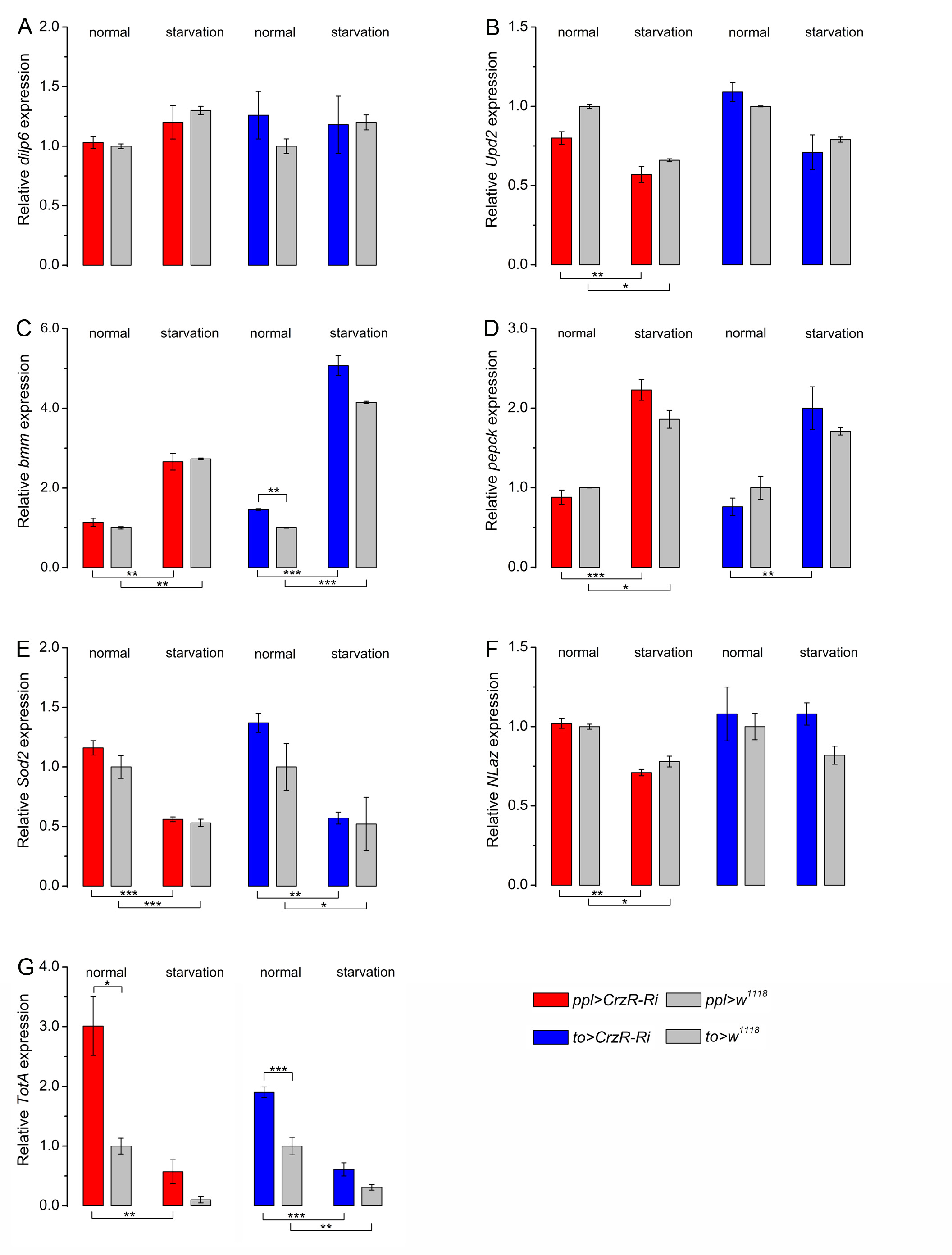

Supplement: Supplementary Figure 6 [file rsob160152supp6.jpg]

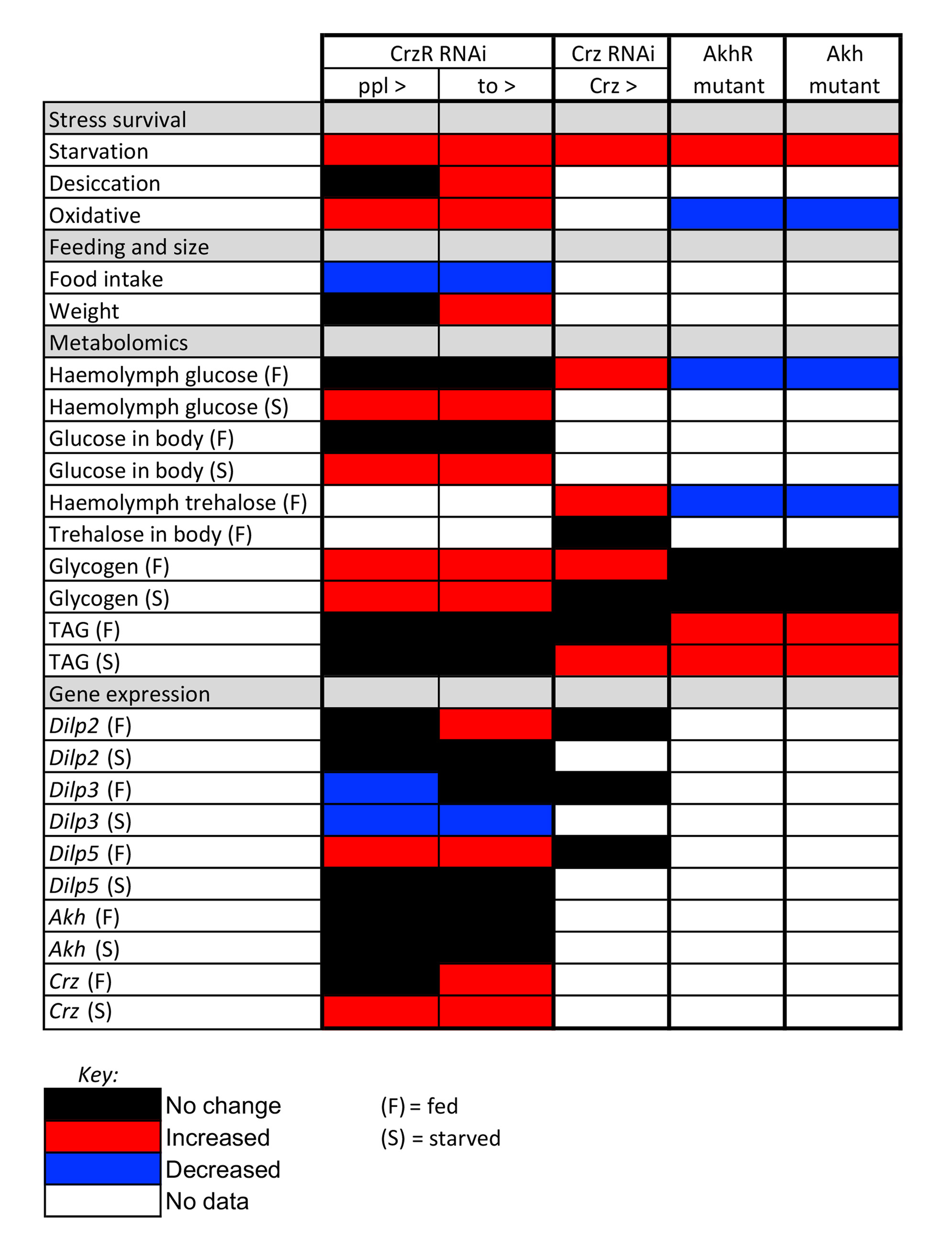

Supplement: Supplementary Figure 7 [file rsob160152supp7.jpg]
