# Supplementary material for: Systemic corazonin signalling modulates stress responses and metabolism in Drosophila
Source: Open Biol. 2016 Nov 3;6(11):160152. doi: 10.1098/rsob.160152 (PMC5133436; doi:10.1098/rsob.160152)
Supplement: Table S1 [file rsob160152supp8.docx]

**Supplemental material files**

**A novel GnRH-like signaling to adipocytes modulates stress responses and metabolism in *Drosophila***

Olga I. Kubrak, Oleh V. Lushchak^1^, Meet Zandawala and Dick R. Nässel^2^

**Table S1.** Primers for qPCR (all displayed 5'–3')^1^

| **Primer** | **Sequence** | **Reference** |
| --- | --- | --- |
| *rp49* F  *rp49* R | ATC GGT TAC GGA TCG AAC AA  GAC AAT CTC CTT GCG CTT CT | [[70](#_ENREF_70)] |
| *Act88* F  *Act88* R | AGG GTG TGA TGG TGG GTA TG  CTT CTC CAT GTC GTC CCA GT | [[31](#_ENREF_31)] |
| *Akh* F  *Akh* R | AGA CCT CCA ACG AAA TGC TG  GTG CTT GCA GTC CAG AAA GAG | [[98](#_ENREF_98)] |
| *dilp2 F*  *dilp2 R* | AGC AAG CCT TTG TCC TTC ATC TC  ACA CCA TAC TCA GCA CCT CGT TG | [[31](#_ENREF_31)] |
| *dilp3 F*  *dilp3 R* | TGT GTG TAT GGC TTC AAC GCA ATG  CAC TCA ACA GTC TTT CCA GCA GGG | [[31](#_ENREF_31)] |
| *dilp5* F  *dilp5* R | GAG GCA CCT TGG GCC TAT TC  CAT GTG GTG AGA TTC GGA GG | [[40](#_ENREF_40)] |
| *dilp6 F*  *dilp6 R* | CCC TTG GCG ATG TAT TTC CCA ACA  CCG ACT TGC AGC ACA AAT CGG TTA | [[70](#_ENREF_70)] |
| *Upd2 F*  *Upd2 R* | CGG AAC ATC ACG ATG AGC GAA T  TCG GCA GGA ACT TGT ACT CG | [[70](#_ENREF_70)] |
| *pepck F*  *pepck R* | TCA ATG GCG AAT CCT GCT AC  TCC TTC ACG TCC ACC TTA TCC | [[98](#_ENREF_98)] |
| *bmm F*  *bmm R* | GGT CCC TTC AGT CCC TCC TT  GCT TGT GAG CAT CGT CTG GT | [[98](#_ENREF_98)] |
| *NLaz F*  *NLaz R* | GGT GAA TGC GGC CAT CAA TC  AAT GGC TGC GTC GGG TAA AA | This study |
| *TotA F*  *TotA R* | AAT TCT TCA ACT GCT CTT ATG TGC  TTT GGA GTC ATC GTC CTG GG | This study |
| *Sod2 F*  *Sod2 R* | CAA ACT GCA AGC CTG GCG  CTC CTC GGC GGC ATT TAG AT | This study |
| *CrzR F*  *CrzR R* | AAT CCG GAC AAA AGG CTG GG  AGG TGG AAG GCA CCG TAG AT | This study |
| *crz F*  *crz R* | GAG GTC CTT TAA CGC CGC AT  TGG CAT TGA AGT CCG AAC CG | This study |

^1^ Unless otherwise stated, primers were designed with the free online software Primer Blast, BLAST database from NIH, Bethesda, Maryland, USA (http:blast.ncbi.nlm.gov/Blast/cgi). For the other primers the corresponding references are shown.
